# The Female Sex Confers Different Prognosis in Heart Failure: Same Mortality but More Readmissions

**DOI:** 10.3389/fcvm.2021.618398

**Published:** 2021-03-05

**Authors:** Raquel López-Vilella, Elena Marqués-Sulé, Rocío del Pilar Laymito Quispe, Ignacio Sánchez-Lázaro, Víctor Donoso Trenado, Luis Martínez Dolz, Luis Almenar Bonet

**Affiliations:** ^1^Heart Failure and Transplant Unit, La Fe University and Polytechnic Hospital, Valencia, Spain; ^2^Cardiology Department, La Fe University and Polytechnic Hospital, Valencia, Spain; ^3^Department of Physiotherapy, University of Valencia, Valencia, Spain; ^4^Centro de Investigación Biomédica en Red Enfermedades Cardiovaculares, CIBERCV, Valencia, Spain; ^5^Department of Medicine, University of Valencia, Valencia, Spain

**Keywords:** heart failure, sex, gender, mortality, morbidity, readmissions, left ventricular ejection fraction

## Abstract

**Introduction:** Heart failure (HF) is a major cause of morbimortality both in men and women. Differences between sex in etiopathogenesis, response to treatment, and quality of care have been found in patients with HF. Females are usually under-represented in clinical trials and there is no solid evidence demonstrating the influence of sex in the prognostic of chronic HF. The primary objective of this study was to analyse the differences in mortality and probability of hospital readmission between males and females with HF. The secondary objective was to compare mortality and probability of hospital readmission by ejection fraction (reduced vs. preserved).

**Methods:** Patients with decompensated HF that were consecutively admitted to a Cardiology Service of a tertiary hospital for 4 years were recruited. *De novo* HF, death during hospitalization, programmed admissions and those patients with moderate left ventricular ejection fraction (LVEF) (40–50%) were discarded. Finally, 1,291 patients were included. Clinical profiles, clinical history, functional status, treatment at admission, first blood analysis performed, readmissions and mortality at follow-up were analyzed and compared. All patients underwent an echocardiographic study at admission. HF with reduced ejection fraction (HFrEF) was considered when left ventricular ejection fraction (LVEF) was <40%, whilst HF with preserved ejection fraction (HFpEF) was considered when LVEF was ≥50%.

**Results:** 716 participants were male (55%). Basal characteristics showed differences in some outcomes. No differences were found in probability of survival among patients with decompensated HF by sex and ejection fraction (*p* = 0.25), whereas there was a clear tend to a major survival in females with HFrEF (*p* < 0.1). Females presented more readmissions when compared to males, independently from the LVEF (females = 33.5% vs. males = 26.8%; *p* = 0.009). Adjusted multivariate analysis showed no association between sex and mortality (HR = 0.97, IC 95% = 0.73–1.30, *p* = 0.86), although there was association between female sex and probability of readmission (OR = 1.37, IC 95% = 1.04–1.82, *p* = 0.02).

**Conclusions:** Sex does not influence mid-term mortality in patients admitted for decompensated HF. Nevertheless, probability of readmission is higher in females independently from LVEF. Thus, it should be considered whether healthcare may be different depending on sex, and a more personalized and frequent care may be recommended in females.

## Introduction

Heart failure (HF) is a major cause of morbimortality both in males and females (1). The incidence is higher in males, although in elders the prevalence is higher in females, due to the fact that females usually have a higher survival rate after the onset of the disease, and as age advances prevalence increases when comparing to males (2–4). Therefore, the total number of patients with HF the in general population is similar in both sexes, or even higher in females (5). In addition, there are also differences by sex in etiopathogenesis of HF, response to treatment and quality of care (5). On the one hand, HF is presented in most cases as a chronic disease with a high rate of comorbidities, some related to sex (6). On the other hand, it should be taken into account that in general females are under-represented in clinical trials and therefore in clinical guidelines (7). It is known that women receive lower average drug doses, show more adverse effects (8) and undergo less frequently therapies related to advanced HF, such as heart transplantation and ventricular assistance (9). Moreover, care process, resource use, and quality of care in patients with HF may be different depending on sex (10).

However, a small number of studies have analyzed evolution and prognosis by sex and by type of HF in detail. No solid evidence about influence of sex on prognosis of HF has been reported, thus it is still a matter of controverse discussion.

The primary objective of this study was to analyse the differences in mortality and probability of hospital readmission between males and females with HF. The secondary objective was to compare mortality and probability of hospital readmission by ejection fraction (reduced vs. preserved).

## Method

**Figure 1:**
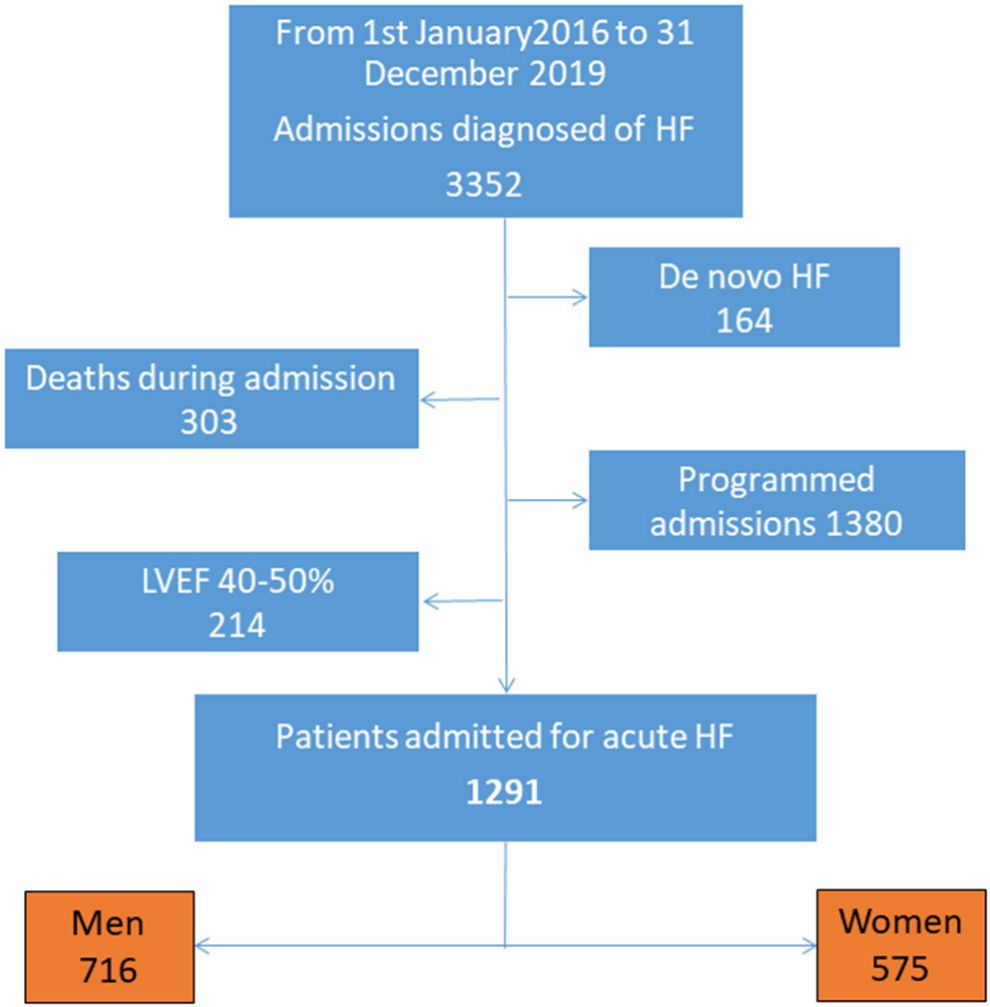
Study flow-chart. HF, Heart failure; LVEF, Left ventricular eyection fraction.

Patients with decompensated HF that were consecutively admitted to a Cardiology Service of a tertiary hospital for 4 years were recruited. This is an ambispective study. *De novo* HF, death during hospitalization, programmed admissions for studies o for therapeutic interventions and those patients with moderate left ventricular ejection fraction (LVEF) (40–50%) were discarded (Figure 1). We decided not to include patients with de novo HF in order to homogenize the sample, so that all patients included in the study are patients with decompensated chronic HF. On the other hand, patients with intermediate ejection fraction were excluded due to their intermediate characteristics between reduced and preserved ejection fraction, and taking into account that it is a less well defined group, in order to make two clear groups of patients. The objective was to select exclusively patients with chronic HF with defined ejection fraction and acute decompensation. Finally, 1,291 patients were included. Clinical profiles, clinical history, functional status, treatment at admission, first blood analysis performed, readmissions and mortality at follow-up were analyzed and compared by sex. All patients underwent an echocardiographic study at admission to assess left ventricular ejection fraction (LVEF). HF with reduced ejection fraction (HFrEF) was considered when LVEF was <40%, whilst HF with preserved ejection fraction (HFpEF) was considered when LVEF was ≥50% (1). The study was approved by the authors' Hospital Research Ethics Committee and all procedures were conducted according to the Declaration of Helsinki. Continuous variables are presented as mean ± SD. Categorical variables are presented as proportions. Univariate comparison was performed using Pearson chi-squared test and t-Student test. Multivariate comparison was performed using Cox regression (survival) and binary logistic regression (readmissions) with death and readmission as dependent variables. Independent variables were those with a significance > 0.05 in the univariate analysis using the intro method. Significance was set at *p* < 0.05. Data were analyzed using SPSS (version 27) and Stata (version 16, number 501606323439).

## Results

### Clinical Characteristics

Univariate analysis showed significant differences when comparing the clinical profile by sex. Differences were conditioned by the different prevalence of underlying heart disease. Therefore, ischemic heart disease was the etiology that most frequently caused HF in men, while in women it was valve disease and hypertension. This fact determines differences in the history of cardiovascular risk factors, percentage of implantation of devices and treatment administered (Table 1).

**Table 1:** Basal characteristics.

	**Women 575**	**Men 716**	***p***
Age (years)	75 ± 12	72 ± 12	0.0001
Previous admissions, *n* (%)	259 (45%)	315 (44%)	0.7
Days admitted at hospital	8.3 ± 6.4	8.7 ± 6.1	0.3
**Underlying heart disease**, ***n*** **(%)**			
Ischemic heart disease	149 (26%)	315 (44%)	0.0001
Non-ischemic cardiomyopathy	52 (9%)	158 (22%)	0.0001
Valve disease	190 (33%)	129 (18%)	0.0001
Congenital heart disease	23 (4%)	7 (1%)	0.0001
Hypertension	132 (23%)	93 (13%)	0.0001
Others	29 (5%)	14 (2%)	0.004
Previous heart surgery, *n* (%)	115 (20%)	158 (22%)	0.4
Hypertension, *n* (%)	460 (80%)	544 (76%)	0.08
Dyslipidemia, *n* (%)	270 (47%)	390 (54%)	0.007
Diabetes mellitus, *n* (%)	253 (44%)	337 (47%)	0.3
Smoker^*^, *n* (%)	75 (13%)	365 (51%)	0.0001
Alcohol^#^, *n* (%)	6 (1%)	64 (9%)	0.0001
Coronary disease	155 (27%)	322 (45%)	0.0001
COPD, *n* (%)	58 (10%)	229 (32%)	0.0001
Obesity (BMI > 30), *n* (%)	63 (11%)	100 (14%)	0.1
Hypothyroidism, *n* (%)	86 (15%)	50 (7%)	0.0001
Atrial fibrilation, *n* (%)	374 (65%)	387 (54%)	0.0001
**NYHA previous to admission**, ***n*** **(%)**			
I	12 (2%)	57 (8%)	0.0001
II	396 (69%)	466 (65%)	0.2
III	155 (27%)	179 (25%)	0.4
IV	12 (2%)	14 (2%)	0.9
SBP (mmHg)	137 ± 25	134 ± 24	0.03
DBP (mmHg)	77 ± 27	78 ± 15	0.4
Heart rate (bpm)	82 ± 21	81 ± 19	0.4
CRT, *n* (%)	12 (2%)	50 (7%)	0.0001
ICD, *n* (%)	17 (3%)	100 (14%)	0.0001
LVEF ≥ 50%	374 (65%)	251 (35%)	0.0001
LVEF <40%	201 (35%)	465 (65%)	0.0001
**Drugs**, ***n*** **(%)**			
Antiplatelets	173 (30%)	308 (43%)	0.0001
Anticoagulant	242 (42%)	272 (38%)	0.1
ACEI/ARB/ARNI	391 (68%)	559 (78%)	0.0001
Beta-blockers	345 (60%)	422 (59%)	0.7
Ivabradine	17 (3%)	50 (7%)	0.001
Diuretics	437 (76%)	437 (61%)	0.0001
MRA	184 (32%)	243 (34%)	0.5
Thiazides	75 (13%)	107 (15%)	0.3
Tolvaptan	23 (4%)	14 (2%)	0.03
Nitrates	35 (6%)	86 (12%)	0.0001
Acetazolamide	12 (2%)	14 (2%)	0.9
Digoxin	46 (8%)	43 (6%)	0.2
Antidiabetics (no iSGLT2)	115 (20%)	236 (33%)	0.0001
SGLTi2	12 (2%)	100 (14%)	0.0001
Potassium supplements	115 (20%)	86 (12%)	0.0001
**Blood analysis**			
Creatinine	1.3 ± 1.1	1.5 ± 1.0	0.001
Sodium	137 ± 4.8	138 ± 4.4	0.2
Potassium	4.4 ± 0.7	4.4 ± 0.6	0.05
NT-ProBNP	8247 ± 6876	8805 ± 7810	0.2
CA125	117 ± 126	119 ± 136	0.2
Troponine T	140 ± 122	176 ± 101	0.0001
Hemoglobin	11.9 ± 1.8	12.4 ± 2.2	0.0001
Uric acid	8.0 ± 2.5	8.2 ± 2.4	0.1

* Current smoker <10 years.

# Alcoholism <1 year.

*ACEI, Angiotensin-converting enzyme inhibitor; ARB, Angiotensin receptor blocker; COPD, chronic obstructive pulmonary disease; CRT, Cardiac resynchronization therapy; DBP, Diastolic blood pressure; ICD, implantable cardioverter defibrillator; iSGLT2, sodium–glucose cotransporter 2 inhibitor; LVEF, Left ventricular ejection fraction; SBP, Sistolic blood pressure*.

### Analysis of Global Morbimortality

**Figure 2:**
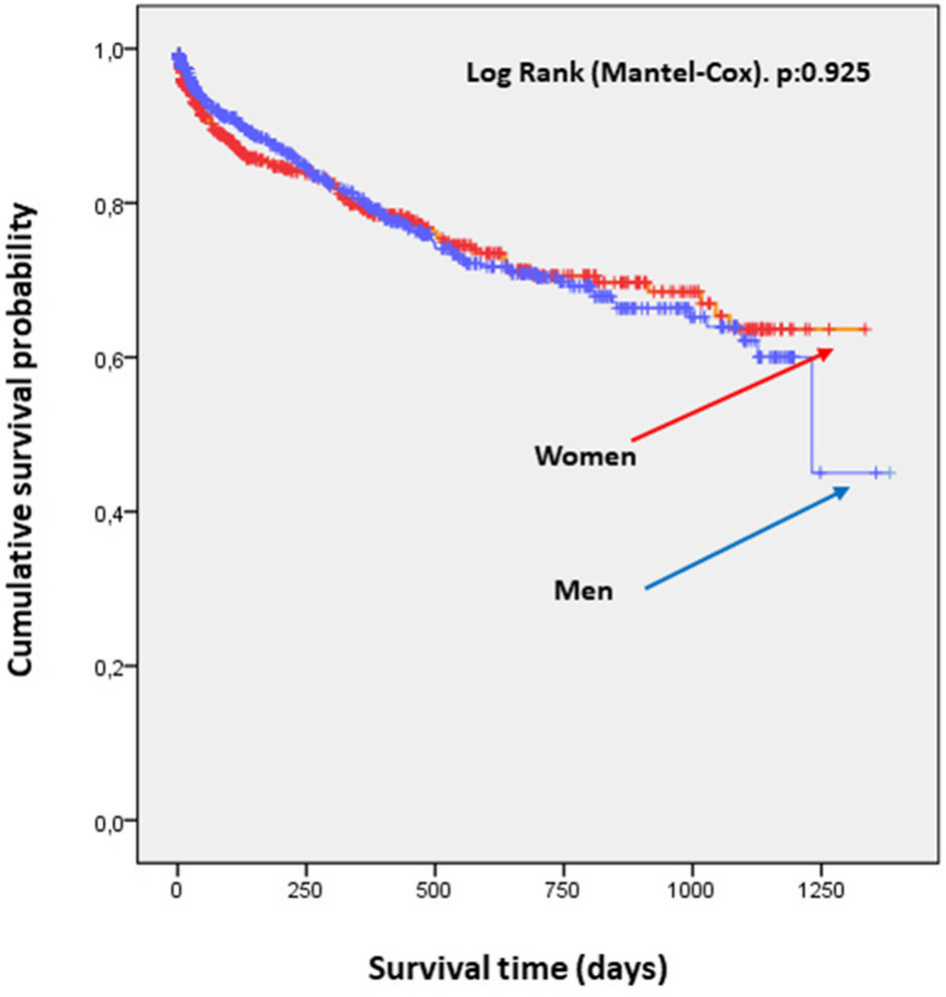
Survival curves by sex. No significant differences were found in probability of survival in patients admitted for decompensated HF by sex.

**Figure 3:**
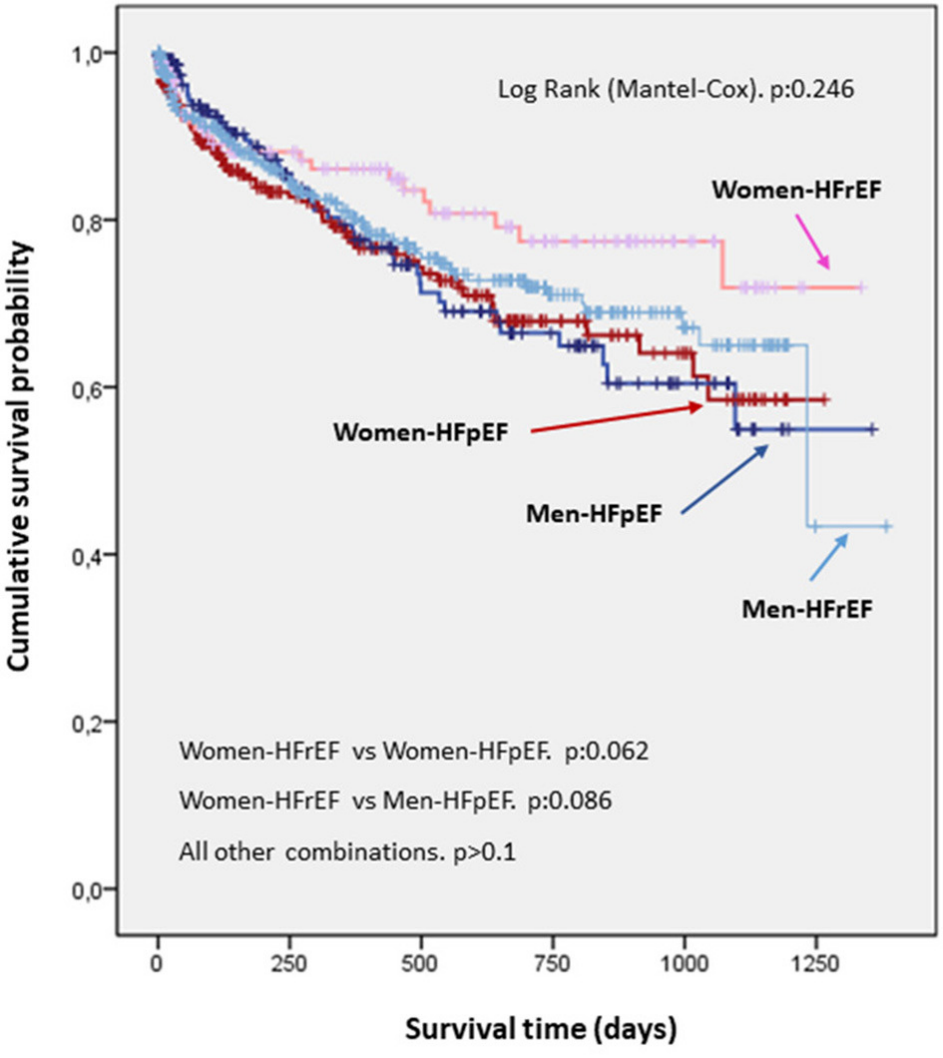
Survival curve by sex and by left ventricular ejection fraction. No differences were observed in probability of survival by sex and by LVEF. Nevertheless, there was a trend for women with HFrEF to have a better prognosis. HFrEF, Heart failure with reduced ejection fraction; HFpEF, Heart failure with preserved ejection fraction.

No differences were found in probability of survival among patients admitted for decompensated HF, independently from sex. The curves were superimposable (Figure 2). There were differences in readmission rates at follow-up between males and females (Figure 3).

### Analysis of Morbimortality by Ejection Fraction

**Figure 4:**
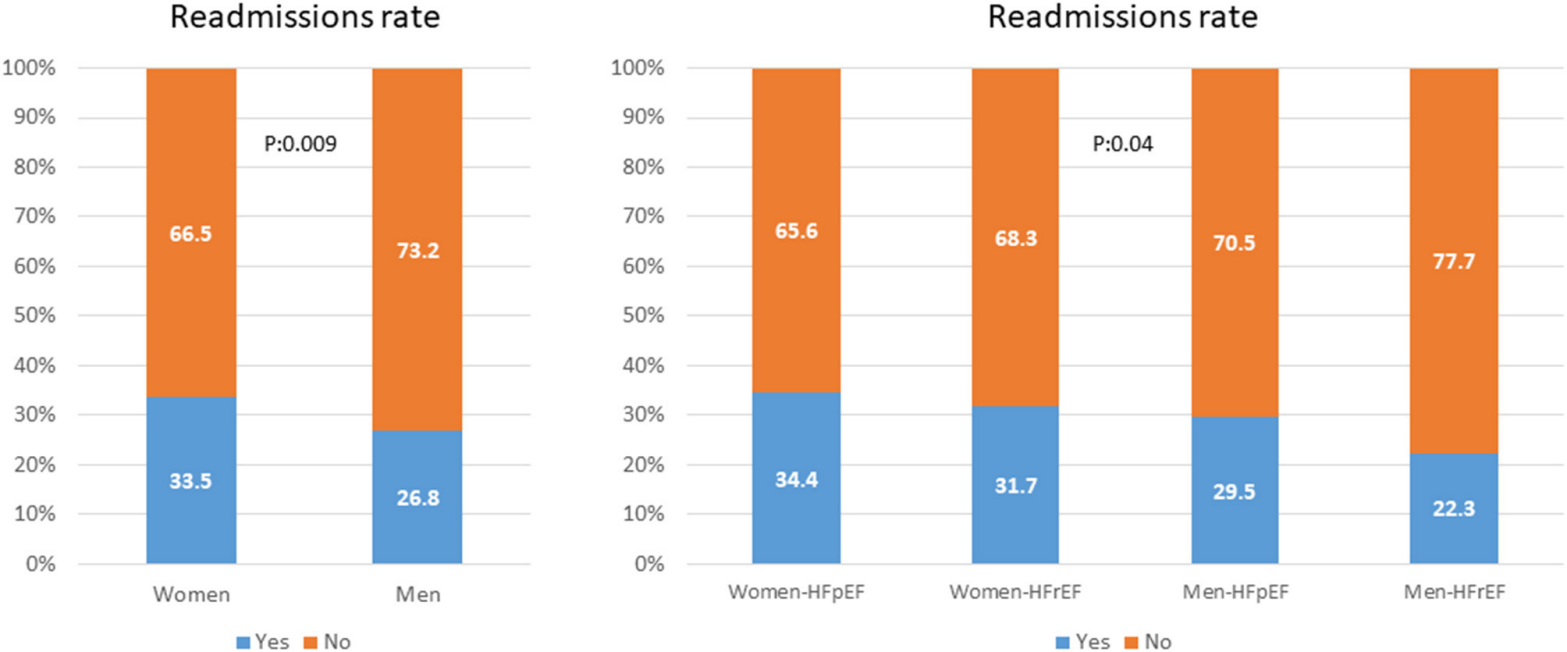
Left: readmission rate between men and women. Right: readmission rate between men and women by LVEF. HFrEF, Heart failure with reduced ejection fraction; HFpEF, Heart failure with preserved ejection fraction.

No differences were found in probability of survival when comparing gender by ejection fraction. Nevertheless, there is an evident trend toward a higher probability of survival in women with decompensated HF and reduced LVEF (Figure 4). There were differences in readmission rate depending on ejection fraction. Thus, women are more frequently readmitted than men, independently from presenting HFrEF or HFpEF (Figure 3).

### Multivariate Analysis

Adjusted multivariate analysis showed no association between sex and mortality. Age and creatinine were related to mortality (Table 2). Adjusted probability of readmission was independently associated to sex and age. LVEF did not show sufficient statistical power to achieve a statistically significant result.

**Table 2:** Multivariate analysis by sex.

	**HR**	**IC95%**	***p***
**Mortality**			
Sex	0.97	0.73–1.30	0,86
Age	1.02	1.01–1.03	0.001
Creatinine	1.32	1.17–1.49	0.0001
	**OR**	**IC95%**	***p***
**Readmission for HF**			
Sex (woman vs. man)	1.37	1.04–1.82	0.02
Age	1.01	1.00–1.02	0.05
LVEF	0.98	0.75–1.29	0.9

Adjusted- analysis to all significant variables in the univariate analysis.

*LVEF, Left ventricular ejection fraction*.

## Discussion

Influence of sex in morbimortality of patients with HF has been subject of debate in the last decade (11, 12). There is an unmet need to assess whether sex differences in comorbidities related to HF require specific management strategies. Differences by sex in clinical profile and LVEF mean that comparison analysis do not allow to extract a sufficiently reliable idea. Therefore, great divergences on the influence of sex on morbimortality of HF are observed in the scientific literature. This study aimed at analyzing whether there were differences by sex in morbimortality in admitted patients with decompensated HF, as well as at follow-up, and whether LVEF was a predictive variable of death or readmissions. Sex does not influence mortality. However, women present a probability of readmission 37% higher with respect to men. On the other hand, it has been stated that LVEF is not independently associated to probability of death nor readmission in patients with decompensated HF.

Basal characteristics of both groups showed differences in the clinical profile of both men and women. In our study, women are older than men, as observed in previous literature, since women tend to develop HF at an older age than men (11, 13–16). Ischemic heart disease is the etiology that most frequently causes HF in men, while in women it is valve disease and hypertension (13, 14, 17, 18). This fact conditions the differences in associated comorbidity and in the history of cardiovascular risk factors: dyslipidemia, smoking, history of alcohol consumption and chronic obstructive pulmonary disease were more frequent in men, in accordance with previous studies (14, 19, 20), whilst othe comorbidities related to HFpEF such as atrial fibrillation and hypothyroidism were more frequent in women. Nevertheless, in our study no greater presence of obesity in females was found, as shown in previous literature (19–22). HFpEF is more frequent in women and represents at least half of the cases of HF in women (13, 17). No differences were found in functional status (NYHA New York Heart Association) II to IV, however, a lower percentage of asymptomatic women was observed in our sample (NYHA I) (14). On the other hand, the percentage of patients with pharmacological treatment for HF is higher in men. Adherence to guidelines in diagnosis treatment of HF is less strict in women than in men, which leads to often insufficient pharmacological treatment with prognostic-modifying drugs for the disease (23, 24). It should be taken into account that this difference could be partially explained by the higher frequency of ischemic heart disease in men, as well as a higher prevalence of HFrEF in men (25). The use of diuretics is more frequent in women, most likely because they are used in the symptomatic control of HF, and it is known that women usually have more severe symptoms than men (5). Women tend to have lower left ventricle end-diastolic volumes at similar left ventricle end-diastolic pressures compared to men. This fact suggests that diastolic dysfunction is an explanation for the paradox of women having more frequent HF symptoms despite frequently preserved left ventricle systolic function (5). Thus, when comparing to men, women have higher rates of dyspnea on exertion, difficulty exercising, and congestion (26–28). Women are less frequently carriers of devices related to HF, both implantable cardioverter defibrillator and cardiac resynchronization therapy (9), despite the fact that some studies have observed that women are more likely to respond favorably to cardiac resynchronization therapy than men (29–31).

One of the most questioned aspects of HF is whether women have a better prognosis than men. Our results support the hypothesis that the survival rate is similar in both sexes, since no significant differences were found in the probability of survival between patients admitted for decompensated HF. Likewise, the adjusted multivariate analysis showed that there is no association between sex and survival, whilst age and creatinine were the only variables associated with mortality. These findings coincide with those obtained in other Spanish registries. In the BADAPIC study (Database of Patients with Heart Failure) (14), carried out mainly in Spanish Departments of Cardiology, similar mortality rates were found in both sexes. Conde-Martel et al. (21) reported, in Departments of Internal Medicine, age-adjusted 1-year mortality rates of 28 and 25% in hospitalized men and women with HF, respectively. In the Olmsted population study, 5-year mortality rates of 59 and 49% were found in outpatient men and women (32, 33). On the other hand, other studies have shown higher survival in women with HF compared to men, however, the effect on sex survival varies according to the characteristics of the cohort. In the I-PRESERVE study (34) in hospitalized patients with preserved LVEF, women had a 20% lower risk of death from cardiovascular and non-cardiovascular events. The MAGGIC meta-analysis (35), with information of 41,949 patients, also showed higher survival for women, suggesting that a lower prevalence of ischemic heart disease, arrhythmias, and sympathetic activation, and better LVEF are protective factors (22, 24).

In our study, no difference was found in the probability of survival when sex was compared by LVEF. However, there was an evident trend toward a higher probability of survival in women with decompensated HF and reduced LVEF. This finding, not described in the previous literature, could be due to the clinical profile of the included women, since in general women with HFpEF associate a greater comorbidity, which frequently determines the prognosis.

It should be noted that readmissions are a growing concern worldwide, since greatly increase the morbidity and mortality of patients and increase the health expenditure of all health systems globally (36). Current patterns of hospital readmission are often associated with organizational factors, such as length of stay, clinical factors, such as age and comorbidities, and factors such as quality of care during admission (37–39). Some authors have focused on sex differences in HF (11, 40–42), although to our best knowledge no study has examined sex differences in relation to readmission rates. Our study has shown significant differences in readmission rate at follow-up between women and men, as well as in the readmission rate depending on LVEF: women are readmitted more frequently than men, independently from having HFpEF or HFrEF. Similarly, the adjusted multivariate analysis confirmed that the adjusted readmission probability was independently associated to gender: the female sex multiplies the readmission probability by 1.37 with respect to men. These data are in line with the trend shown in previous studies (14, 43–46) that observed although the mortality of women and men with HF is similar, the readmission rate for HF is higher in women in specialized HF clinics. These results may be associated with previously described differences in pharmacological treatment. A meta-analysis found more articles reporting that men with HF had significantly higher readmission rates compared to women (47). The effect of sex on readmission may depend on the length of follow-up, with a longer duration of follow-up favoring higher readmission rates among men. Thus, Hoang-Kim et al. (47) reported that the readmission rate for men was higher when the duration of follow-up was >1 year. In contrast, women were more likely to experience higher readmission rates than men when the time to event was <1 year. Consequently, possibly future studies should consider different time horizons in their designs.

One of the most important limitations of previous studies is the lack of data regarding LVEF, data that have been included in this analysis, given the differences by sex in the prevalence of HFrEF vs. HFpEF. Differentiating the LVEF allows us to analyze the effect of this relevant clinical variable in the evaluation of sex differences in the treatment and prognosis of HF.

The limitations of this study are those related to the patient databases However, this database is filled prospectively during the admission of the patient, so clinical data have a very high reliability. In addition, echocardiographic studies are performed at each admission so that HF classification does not have a temporal cadence with admission. On the other hand, the clinical impact of this work is high as it is a study with a large number of patients that demonstrates equality of sexes in terms of mortality, but with a greater number of readmissions in women during follow-up, independently from the type of HF.

## Conclusions

Sex does not influence mid-term mortality in patients admitted for decompensated HF. Nevertheless, probability of readmission is higher in females independently from LVEF. Thus, it should be considered whether health strategies may be different depending on sex, and a more personalized and frequent healthcare may be recommended in females.

## Data Availability Statement

The raw data supporting the conclusions of this article will be made available by the authors, without undue reservation.

## Ethics Statement

The studies involving human participants were reviewed and approved by Instituto de Investigación Sanitaria La Fe. The patients/participants provided their written informed consent to participate in this study.

## Author Contributions

RL-V: data collection, data analysis, writing results, writing the manuscript, translation, and revision of the final manuscript. EM-S: data collection, translation and revision of the final manuscript. RL: data collection and writing the manuscript and revision of the final manuscript. IS-L, VD, and LM: writing the manuscript and revision of the final manuscript. LA: data analysis, writing results, writing the manuscript and translation and revision of the final manuscript. All authors contributed to the article and approved the submitted version.

## Conflict of Interest

The authors declare that the research was conducted in the absence of any commercial or financial relationships that could be construed as a potential conflict of interest.

## Abbreviations

HF: Heart failure
HFrEF: Heart failure HF with reduced ejection fraction
HFpEF: Heart failure HF with preserved ejection fraction
LVEF: Left ventricular ejection fraction.
